# CesL Regulates Type III Secretion Substrate Specificity of the Enteropathogenic *E. coli* Injectisome

**DOI:** 10.3390/microorganisms9051047

**Published:** 2021-05-13

**Authors:** Miguel Díaz-Guerrero, Meztlli O. Gaytán, Eduardo Soto, Norma Espinosa, Elizabeth García-Gómez, Arely Marcos-Vilchis, Angel Andrade, Bertha González-Pedrajo

**Affiliations:** 1 Departamento de Genética Molecular, Instituto de Fisiología Celular, Universidad Nacional Autónoma de México, Ciudad de México 04510, Mexico; madiaz@ifc.unam.mx (M.D.-G.); meztlli.gaytan@gmail.com (M.O.G.); jose.soto@yale.edu (E.S.); nespino@ifc.unam.mx (N.E.); amarcos@ifc.unam.mx (A.M.-V.); 2 Unidad de Investigación en Reproducción Humana, Consejo Nacional de Ciencia y Tecnología-Instituto Nacional de Perinatología, Ciudad de México 11000, Mexico; egarciag@conacyt.mx; 3 Departamento de Microbiología, Facultad de Medicina, Universidad Autónoma de Nuevo León, Monterrey, Nuevo León 64460, Mexico; angel.andradet@uanl.edu.mx

**Keywords:** type III secretion system, EPEC, secretion hierarchy, CesL, gatekeeper complex

## Abstract

The type III secretion system (T3SS) is a complex molecular device used by several pathogenic bacteria to translocate effector proteins directly into eukaryotic host cells. One remarkable feature of the T3SS is its ability to secrete different categories of proteins in a hierarchical manner, to ensure proper assembly and timely delivery of effectors into target cells. In enteropathogenic *Escherichia coli*, the substrate specificity switch from translocator to effector secretion is regulated by a gatekeeper complex composed of SepL, SepD, and CesL proteins. Here, we report a characterization of the CesL protein using biochemical and genetic approaches. We investigated discrepancies in the phenotype among different *cesL* deletion mutants and showed that CesL is indeed essential for translocator secretion and to prevent premature effector secretion. We also demonstrated that CesL engages in pairwise interactions with both SepL and SepD. Furthermore, while association of SepL to the membrane does not depended on CesL, the absence of any of the proteins forming the heterotrimeric complex compromised the intracellular stability of each component. In addition, we found that CesL interacts with the cytoplasmic domains of the export gate components EscU and EscV. We propose a mechanism for substrate secretion regulation governed by the SepL/SepD/CesL complex.

## 1. Introduction

Enteropathogenic *Escherichia coli* (EPEC) is a causal agent of human intestinal disease generating acute watery diarrhea in children [1]. EPEC belongs, together with enterohemorrhagic *E. coli* (EHEC), *E. albertii* and *Citrobacter rodentium* (CR), to a group of enteric pathogens (AE pathogens) able to induce an attaching and effacing histological lesion (AE lesion), characterized by the destruction of brush border microvilli, remarkable cytoskeletal alterations and intimate adherence of bacteria to the epithelial cell membrane [2,3,4]. A central element to EPEC pathogenicity is a type III secretion system (T3SS) or injectisome, which translocates effector proteins into the enterocyte cytosol [5,6]. The T3SS in AE pathogens is encoded on a chromosomal pathogenicity island known as locus of enterocyte effacement (LEE), which comprises 41 genes arranged into seven operons and four individual transcriptional entities [7,8,9]. Seven of the translocated effectors are encoded within the LEE, while the genes for around 16 others (named Nle, for non-LEE encoded effectors) are distributed elsewhere in the chromosome [10,11].

The T3SS is a supramolecular assemblage of about 20 different proteins organized into a multiring basal body that spans both membranes and the periplasm, a transmembrane export apparatus housed within the basal body at the inner membrane (IM), a set of cytosolic components, and an external conduit for secreted proteins [5,12]. Bacterial flagella are assembled by an evolutionarily related T3SS and several components of the flagellum share sequence and structural similarities to their injectisome counterparts [13,14]. In addition, it has recently been shown that the T3SS export apparatus also serves to assemble membranous surface structures called nanotubes, through which bacteria extract nutrients from the infected host cell [15].

In EPEC, the export apparatus is formed by the proteins EscR, EscS, EscT, EscU, and EscV (SctRSTUV in the unified T3SS nomenclature [16,17]) that together with the cytosolic ATPase complex formed by three proteins, EscN, EscL, EscO (SctNLO) and the sorting platform composed of EscQ, EscL and EscK (SctQLK), are crucial for secretion, having an important role in type III substrate docking and recognition as well as in energy fueling [5,18,19,20,21]. Moreover, based on what is known in other virulence T3S systems and in the flagellar T3SS (fT3SS), the core export apparatus proteins SctRST assemble as a helical structure above the IM, and the transmembrane domain of SctU wraps around this core while its C-terminal portion resides in the cytoplasmic side [22]. SctV is a polytopic IM protein with a large cytoplasmic C-terminal domain arranged as a toroidal nonameric ring, which is proposed to form an entry gate for T3 substrates [23,24,25].

T3 effectors travel through an extracellular continuous channel formed by a needle (a helical polymer of EscF subunits) a filament (a polymer of EspA subunits) and a translocation pore (formed by subunits of EspB and EspD proteins), which allows the passage of effector proteins through the host plasma cell membrane [26,27,28,29]. Multiple T3S substrates require the assistance of cognate chaperones for stabilization, to maintain a competent secretion conformation, for targeting to the export apparatus, to establish a secretion hierarchy and for proper translocation [30,31,32].

T3SS assembly and effector translocation are strictly regulated processes so that protein secretion is synchronized with the different stages of injectisome biogenesis. After the Sec-dependent assembly of the membrane rings and export apparatus, plus integration of the sorting platform and ATPase complex, type III mediated secretion initiates with the delivery of early substrates that form the inner rod and needle, followed by middle substrates or translocators, and lastly by late substrates or effectors [33,34]. The orderly secretion of these three different classes of proteins is achieved by two molecular switches that guarantee a precise secretion hierarchy.

The first substrate specificity switch, from early to middle substrates, is controlled by two proteins, EscU that belongs to the SctU family of proteins (YscU/SpaS/Spa40) and EscP of the SctP family (YscP/InvJ/Spa32). The ruler protein EscP is secreted until the needle reaches its final length, then, a productive interaction of EscP with the autocleaved form of EscU stops the secretion of early substrates and promotes, together with the second secretion switch, an efficient secretion of translocators [35,36]. The second molecular switch, from middle to late substrates, is regulated by proteins referred to as gatekeepers, SctW in the unified nomenclature (e.g., SepL, InvE, YopN-TyeA, SsaL, MxiC and PopN-Pcr1), and occurs once the translocon has been assembled and when the injectisome establishes contact with the eukaryotic cell [12]. Upon host cell contact, by means of an incompletely understood mechanism, an activation signal is transmitted through the needle-filament to the cytosolic components and the gatekeeper protein is either secreted/translocated, degraded, or dissociated from an acceptor site for effectors, thereby allowing the secretion of late substrates [37,38,39,40,41,42,43,44,45,46,47]. Indeed, it has been shown that some of the gatekeeper proteins regulate secretion substrate specificity by interacting with a component of the SctV protein family (EscV/SsaV/MxiA/PcrD), either altering its conformation to differentially recognize middle or late substrates or blocking a binding site for effectors [48,49,50,51,52]. Furthermore, many gatekeeper proteins promote the secretion of middle substrates, thus deletion mutants of the corresponding genes drastically diminished or abolished translocator secretion [39,41,50,53,54].

In addition, some of the gatekeepers in different T3S systems function as complexes with proteins proposed to be acting as chaperones [42,44,55,56]. In the case of AE pathogens, the SepD protein forms a complex with SepL, and deletion mutants in both *sepD* and *sepL* exhibited an abolished secretion of translocators and hypersecretion of effectors, so the complex was proposed to control the secretion hierarchy between translocators and effectors [36,54,57,58]. Later, it was shown that the SepL–SepD complex associates with a third component, CesL, which was predicted to be a chaperone for SepL [59]. However, a direct interaction between these two proteins has not been proven and the function of CesL has not been extensively studied. It is also intriguing that distinct secretion phenotypes for *cesL* deletion mutants have been reported among AE pathogens [48,54,60]. Besides, SepL has been found to interact in a calcium-dependent manner with EscP, the molecular ruler of the first substrate specificity switch, in order to prevent premature effector secretion. The model proposes that once the translocation pore has been formed, a drop in calcium concentration disrupts the EscP–SepL interaction, enabling the secretion of late substrates [61]. The most recent mechanism reported for the functioning of the second molecular switch involves a SepD–SepL complex with the EscV export gate nonameric ring, rendering a high-affinity receptor for translocator-chaperone substrates and concurrently hindering the effector-chaperone access to the gate. Then, after the cell contact-dependent signal is transduced through the injectisome, SepD and SepL could dissociate from EscV to allow effector secretion [48].

In this work we aimed to characterize the role of CesL in the substrate secretion hierarchy within the heterotrimeric SepL/SepD/CesL complex. We propose a model that includes a functional interconnection between the two molecular switches for regulating translocator to effector secretion.

## 2. Materials and Methods

### 2.1. Bacterial Strains and Growth Conditions

Bacterial strains and plasmids used in this study are listed in Table 1. *E. coli* strains were aerobically grown at 37 °C in Lysogeny Broth (LB) with constant shaking (250 rpm). When needed, bacterial cultures were supplemented with the appropriate antibiotics at the following concentrations: ampicillin, 100 µg/mL; chloramphenicol, 25 µg/mL; kanamycin, 50 µg/mL; streptomycin, 50 µg/mL; tetracycline, 20 µg/mL.

### 2.2. Construction of EPEC Null Mutants and Tagged Strains 

The oligonucleotides used in this study are listed in Table 2. The non-polar mutant of *cesL* (Δ*cesL*) was constructed using the λ-Red recombination system [68]. Briefly, the kanamycin resistance cassette was amplified from plasmid pKD4 using primers delcesLSRS_Fw and delcesL_Rv (Table 2). The resulting PCR product was electroporated into EPEC wild-type (WT), Δ*sepD* or Δ*sepL* strains carrying plasmid pKD46. For the *C. rodentium* deletion mutant (Δ*cesL*_2_::km) the kanamycin resistance cassette was amplified from pKD4 using primers delcesL_Fw and delcesL_Rv. Deletion mutants were selected on LB plates containing kanamycin at 37 °C. The kanamycin cassette was excised from the chromosome of the Δ*cesL*_2_::km mutant using the FLP recombinase encoded in the pFLP2 plasmid [70]. To generate the *sepL*-FLAG Δ*cesL* strain, the kanamycin cassette from the *sepL*-FLAG strain was excised and the *cesL* deletion was obtained as mentioned above. Chromosomal epitope tagging was performed as previously described [69]. The EPEC strains expressing a 3×FLAG-tagged version of *sepL* (*sepL*-FLAG), or a 3×FLAG-tagged version of *cesL* (*cesL*-FLAG), were constructed with the λ-Red recombination strategy using plasmid pSUB11 as a template for amplification of the 3×FLAG-kanamycin cassette [69]. The primers used to tag *sepL* and *cesL* on the chromosome were sepL-3FLAG_Fw/sepL-3FLAG_Rv and cesL-3FLAG_Fw/cesL-3FLAG_Rv, respectively. All genetic modifications were verified by PCR. Proper expression of the FLAG-tagged proteins was confirmed by immunoblotting.

### 2.3. Plasmid Construction

Restriction enzymes, Q5 DNA Polymerase and T4 DNA ligase were obtained from New England Biolabs and used according to the manufacturer’s instructions. PCR reactions were performed using EPEC genomic DNA as template and the indicated primers. All plasmids used in this work are listed in Table 1. Plasmids pMEpL and pMGADpL were generated by subcloning *sepL* from pMTpL, into the NdeI/BamHI restriction sites of the pET19b and pGADT7 vectors, respectively. Plasmids pAEpD, pMATpD and pOGBpD, were constructed by subcloning *sepD* from pATpD, into the NdeI/BamHI sites of the pET19b, pACTrc and pGBKT7 vectors, respectively. Plasmids pMTcL, pMATcL and pMGADcL, were made by subcloning *cesL* from pMEcL, into the NdeI/BamHI sites of pTrc99A_FF4, pACTrc and pGADT7 vectors, respectively. Plasmid pMTHcL was generated by subcloning *his-cesL* from pMEcL, into the NcoI/BamHI sites of pTrc99A vector.

PCR amplification of *sepL* truncated versions *sepL*_Δ30_ and *sepL*_Δ75_, was achieved using the primer pairs sepLNdeIΔ30_Fw/sepLBamHIRv and sepLNdeIΔ75_Fw/sepLBamHI_Rv, respectively. A *sepL* in-frame deletion lacking codons 240 to 282 (*sepL*_Δ81-94_) was constructed by overlapping PCR. The first round of PCR was performed with primer pairs sepLNdeI_Fw/sepLΔ81-94-A_Rv and sepLΔ81-94-B_Fw/sepLBamHI_Rv, and the resulting amplicons were purified and used as template for a second round of PCR using the primer pair sepLNdeI_Fw/sepLBamHI_Rv. Purified amplicons *sepL*_Δ30_, *sepL*_Δ75_ and *sepL*_Δ81-94_ were cloned into the NdeI/BamHI sites of pTrc99A_FF4 or pET19b plasmids to generate pMATpL_Δ30_, pMEpL_Δ75_, and pMEpL_Δ81-94_, respectively. Plasmids pMATpL_Δ81-94_ and pOGADpL_Δ81-94_ were generated by subcloning *sepL*_Δ81-94_ from pMEpL_Δ81-94_ into the NdeI/BamHI sites of pACTrc and pGADT7, respectively.

To generate bicistronic constructs, plasmids pMEcL, pAEpD, pMEpL or pMEpL_Δ75_, were digested with XbaI/PstI restriction enzymes. The resulting products *his-cesL*, *his-sepD*, *his-sepL*, and *his-sepL*_Δ75_ were subcloned into the XbaI/PstI sites of pMTpL or pATpD, yielding plasmids pMTBISpLcL, pMTBISpLpD, pMTBISpDpL and pMTBISpDpL_Δ75_, respectively.

Plasmids encoding *cesL*_C__Δ10_ (lacking codons 107-117), were constructed by PCR using the primer pair cesLNdeI_Fw/cesL_C__Δ10__Rv. The resulting amplicon was cloned into the NdeI/BamHI sites of pACTrc or pGADT7 plasmids to generate pMATcL_C__Δ10_ and pMGADcL_C__Δ10_, respectively. To construct CesL N-terminal MBP-tagged versions, full-length *cesL* or *cesL*_C__Δ10_ were amplified by PCR with primer pairs cesLBamHI_Fw/cesLHindIII_Rv and cesLBamHI_Fw/cesLMALPstI_Rv, respectively. Purified amplicons were cloned into the BamHI/HindIII or BamHI/PstI sites of pMAL-c2X plasmid to generate pMLcL and pMLcL_C__Δ10_, respectively. The C-terminal 2HA-tagged version of CesL (CesL-HA) was generated by PCR amplification of the *cesL* coding region including its native ribosome-binding site with the primer pair cesLHA_Fw/cesLHA_Rv. The purified amplicon was cloned into the HindIII/XhoI sites of pTOPO-2HA plasmid to generate pMHcL. Plasmid pMATcL2HA was constructed by subcloning *cesL-2HA* from pMHcL into the HindIII/BamHI sites of the pACTrc vector.

To create the bacterial two-hybrid constructs, the coding regions of *sepD*, *tir*, and *cesL* were amplified by PCR using the primer pairs sepDXhoI_Fw/sepDKpn_Rv, tirXhoI_Fw/tirKpn_Rv and cesLXhoI_Fw/cesLKpnI_Rv, respectively. The purified amplicons were cloned into the XhoI/KpnI sites of pSR658 vector, to create pMR58pD, pMR58tir and pMR58cL, respectively. Additionally, *sepL* and *cesT* were amplified by PCR with the primer pairs sepLBamHI_Fw/sepLXhoIstop_Rv and cesTBamHI_Fw/cesTKpnI_Rv, respectively. Purified amplicons were cloned into the BamHI/KpnI sites of pSR659 vector to create pMR59pL and pMR59cT, respectively. Plasmid pMR59pD was generated by subcloning *sepD* from pMR58pD into the XhoI/KpnI sites of pSR69 vector.

All constructs resulting from PCR amplification were verified by DNA sequencing at the Instituto de Fisiología Celular, UNAM.

### 2.4. Protein Solubility Assay 

Plasmids transferred from *E. coli* into *Salmonella* SJW1368 were first transformed into *Salmonella* JR501, which is a restriction-deficient modification-proficient strain [73]. *Salmonella* SJW1368 harboring plasmids pMTBISpDcL, pMTBISpLcL or pMTBISpDcL, in addition to plasmid pMATpL, was grown at 37 °C until an OD_600_ of 0.6–0.8 was reached. Protein expression was induced with 0.1 mM isopropyl-β-D-thiogalactopyranoside (IPTG) for 4 h at 30 °C. Cell cultures were harvested by centrifugation at 8000× *g* for 10 min. The harvested cells were resuspended in 10 mL of buffer (20 mM Tris-HCl pH 7.4, 500 mM NaCl), lysed by sonication and centrifuged at 27,000× *g* for 30 min at 4 °C to separate the soluble and insoluble fractions. The insoluble fraction (pellet) was resuspended in the same buffer and volume as that used for the soluble fraction. Buffer 1× SDS-PAGE was added to protein samples from both fractions, normalized according to the OD_600_ of the respective bacterial culture.

### 2.5. Purification of Trimeric Complexes

*Salmonella* SJW1368 cells co-transformed with plasmids pMTBISpDpL and pMATcL, pMTBISpLpD and pMATcL, pMTBISpDcL and pMATpL, pMTBISpDcL and pMATpL_∆30_ or pMTBISpDpL_∆75_ and pMATcL were grown in 200 mL of LB to an OD_600_ of 0.6–0.8. At this point, protein production was induced by addition of 0.1 mM IPTG and bacterial growth was continued for 4 h at 30 °C. Cells were collected by centrifugation at 8000× *g* for 10 min and resuspended in 10 mL of lysis buffer (20 mM Tris-HCl pH 7.4, 500 mM NaCl, 10 mM imidazole) supplemented with 1 mM phenylmethylsulfonyl fluoride (PMSF). Cells were lysed by sonication and then centrifuged at 27,000× *g* for 30 min at 4 °C. The cleared cell lysate was loaded onto a column containing 200 μL of Ni-NTA agarose beads (Qiagen). Coupled agarose beads were washed three times with 10 mL of lysis buffer with increasing concentrations of imidazole (10 mM, 30 mM and 60 mM). Finally, bound proteins were eluted with three column volumes of lysis buffer containing 400 mM imidazole.

### 2.6. Pull-Down Assays 

*E. coli* BL21 (DE3) pLysS (BDP) cells carrying plasmids pMAL-c2X, pMLcL, pMLcL_cΔ10_, pATpD, pMTpL, pKEeV_C_, pJEeU_C_ or pKEeD_N_ were cultured in 100 mL of LB supplemented with 0.1% glucose at 37 °C until an OD_600_ of 0.6–0.8 was reached. Then, IPTG was added to a final concentration of 0.3 mM for pMAL-c2X-based constructions or 0.1 mM for pTrc99A_FF4- and pET19b-based constructions, and growth was continued for 4 h at 30 °C. Cells were collected by centrifugation at 8000× *g* for 10 min, resuspended in 10 mL of lysis buffer (50 mM Tris-HCl pH 7.4, 200 mM NaCl, 1 mM EDTA, 1 mM PMSF) and lysed by sonication. The cell lysates were cleared by centrifugation (27,000× *g* for 30 min). Cleared lysates containing MBP, MBP-CesL or MBP-CesL_CΔ10_ were incubated with 200 µL of amylose resin (New England Biolabs) for 30 min at 4 °C with gentle shaking. The amylose resin coupled to MBP or MBP-tagged proteins was loaded onto a column and washed with 5 mL of lysis buffer. At this point, the cleared cell lysate containing SepD, SepL, His-EscV_C_, His-EscU_C_ or His-EscD_N_ was loaded into the column. Unbound proteins were washed three times with 5 mL of column buffer (50 mM Tris-HCl pH 7.4, 500 mM NaCl). In the case of the MBP-CesL_CΔ10_/SepD and MBP-CesL/SepD pull-downs, the final wash contained 1M NaCl. Proteins were eluted three times with 200 µL of column buffer containing 20 mM maltose.

To test the interaction between His-SepL, His-SepL_Δ81-94_ or His-SepL_Δ75_ and SepD, BDP cells harboring plasmids pMEpL, pMEpL_Δ81-94_, pMEpL_Δ75_ or pATpD were grown in 200 mL of LB at 37 °C to an OD_600_ of 0.6–0.8. Then, IPTG was added to a final concentration of 0.1 mM and bacterial growth was continued for 4 h at 30 °C. Cells were collected by centrifugation at 8000× *g* for 10 min, resuspended in lysis buffer (20 mM Tris-HCl pH 7.4, 500 mM NaCl, 10 mM imidazole and 1 mM PMSF) and lysed by sonication. The cell lysates were cleared by centrifugation (27,000× *g* for 30 min). Cleared lysates containing His-SepL, His-SepL_Δ81-94_ or His-SepL_Δ75_ were incubated with 200 µL of Ni-NTA resin for 30 min at 4 °C with gentle shaking. The protein-coupled resin was loaded into a column and washed with 5 mL of lysis buffer. Subsequently, the cleared cell lysate containing SepD was loaded into the column. Unbound proteins were washed three times with lysis buffer containing 30 mM imidazole. Proteins were eluted three times with 200 µL of lysis buffer containing 400 mM imidazole.

### 2.7. Bacterial Two-Hybrid Assay

This assay is based on the modular nature of the transcriptional repressor LexA. This protein comprises an N-terminal DNA binding domain (DBD) and a C-terminal dimerization domain, and it binds to an operator site only as a dimer. The bait protein is fused to a mutant LexA DBD (LexA408) which binds to a mutant operator sequence and the prey protein is fused to a wild-type LexA DBD (LexAWT). Heterologous interactions between the proteins tested reconstitute the LexA heterodimer, which binds to a hybrid operator site, repressing the expression of the *lacZ* reporter gene [62]. The reporter *E. coli* SU202 strain was co-transformed with pSR658- and pSR659-based constructs (Table 1). Co-transformants were grown overnight in LB containing ampicillin, tetracycline and 1 mM IPTG. Overnight cultures were used to inoculate fresh LB supplemented with 1 mM IPTG and the appropriate antibiotics, and bacterial growth was continued until an OD_600_ of 0.4–0.6 was reached. The β-galactosidase activity was determined as described by Miller [74]. Briefly, 100 μL aliquots of each culture were mixed with 900 µL of buffer Z (60 mM Na_2_HPO_4_ •7H2O, 40 mM NaH_2_PO_4_ •H_2_O, 100 mM KCl, 1 mM MgSO_4_ •7H2O, 50 mM β-mercaptoethanol) pH 7.0. One drop of chloroform was added to the mixture and immediately vortexed for 30 s to lyse the cells. Then, 200 µL of ortho-Nitrophenyl-β-galactoside (ONPG) (4mg/mL) were added and tubes were incubated at 30 °C for 30 min. The reaction was stopped with 500 µL of 1M NaCO_3_ and the OD_420_ and OD_550_ was recorded. The Miller units were calculated as previously described [74]. Assays were performed in triplicate. Collected data were analyzed by a two-tailed Student’s *t*-test using the GraphPad Prism software.

### 2.8. Yeast Two-Hybrid Assay

The Matchmaker GAL4 two-hybrid system (Clontech) was used to evaluate protein–protein interactions in yeast as previously described [20]. *Saccharomyces cerevisiae* strains PJ69-4α (MAT-α) and PJ69-4a (MAT-a) were transformed with pGBKT7 or pGADT7-based constructs, respectively. Transformants were plated onto minimal synthetic-dropout medium lacking tryptophan (SD-Trp) or leucine (SD-Leu). Mating was carried out by co-incubating the transformed MAT-α and MAT-a strains in Yeast Extract–Peptone–Dextrose (YPD) medium for 5 h at 30 °C with shaking. Cells harboring both plasmids were selected in SD-Trp-Leu plates. Protein interactions were selected by 10-fold serial dilutions spotted (3 µL of each dilution) onto SD-Trp-Leu-His medium. Plates were incubated for 3 to 9 days.

### 2.9. Type III Secretion Assay

EPEC strains were grown overnight at 37 °C in LB medium supplemented with the appropriate antibiotics. Overnight cultures were used to inoculate (1:100 dilution) pre-equilibrated DMEM at 37 °C in a 5% CO_2_ atmosphere. Bacterial growth was continued under static conditions and the abovementioned parameters to an OD_600_ of 0.7–0.8. Cells were harvested by centrifugation at 8000× *g* for 10 min and the cellular pellet was resuspended in 1× SDS-PAGE sample buffer. The proteins in the supernatant were precipitated overnight at 4 °C with 10% *v*/*v* trichloroacetic acid, and centrifuged at 18,100× *g* for 30 min. The resulting protein pellet was resuspended in 1× SDS-PAGE sample buffer containing 10% *v*/*v* saturated Tris. Protein concentration in all samples was normalized according to each culture OD_600_ value.

### 2.10. Cell Fractionation

EPEC strains from overnight LB cultures were diluted 1:100 in 50 mL of pre-equilibrated DMEM and grown statically at 37 °C in a 5% CO_2_ atmosphere to an OD_600_ of 0.7–0.8. Cells were collected by centrifugation at 8000× *g* for 10 min, washed with 10 mL of phosphate-buffered saline, and centrifuged as described above. Bacterial cells were resuspended in 10 mL of 20 mM Tris-HCl pH 7.4 supplemented with 1 mM PMSF and disrupted by sonication. Samples were then centrifuged twice at 8000× *g* for 20 min to remove intact cells each time. The supernatant was ultracentrifuged at 90,400× *g* for 1 h at 4 °C to separate the cytoplasmic fraction (supernatant) from the membrane fraction (pellet). The membrane fraction was washed with 10 mL of cold Tris-HCl pH 7.4, ultracentrifuged as above and resuspended in 1 mL of Tris-HCl pH 7.4. Total protein content was quantified by Lowry method [75]. To analyze proteins by immunodetection, 6 µg of total protein were loaded and separated by SDS-PAGE.

### 2.11. Immunoblotting

Proteins were resolved on a 15% SDS-PAGE and transferred onto PVDF or nitrocellulose membranes, which were then blocked overnight with 5% *w*/*v* non-fat milk in TBS-T (Tris-buffered saline with 0.1% *v*/*v* Tween 20). The following antibodies were used for immunoblotting: anti-EspA, anti-EspB, anti-EspF, anti-Map, anti-SepL, anti-SepD, anti-EscQ, anti-EscJ, anti-CesL or anti-Tir polyclonal antibodies; as well as anti-DnaK (Enzo Life Sciences), horseradish peroxidase (HRP)-conjugated anti-FLAG (Sigma), anti-HA (Thermo Scientific) or anti-HIS (Santa Cruz Biotechnology) monoclonal antibodies. When required, membranes were probed with a secondary antibody (HRP-conjugated anti-rabbit or anti-mouse antibodies). Protein detection was carried out using the Western Chemiluminescent HRP Substrate kit (Millipore).

### 2.12. Protein Stability Assay

EPEC strains were grown overnight in LB and diluted 1:100 into 20 mL of pre-equilibrated DMEM. Bacterial growth was continued under static conditions at 37 °C in a 5% CO_2_ atmosphere until an OD_600_ of 0.7–0.8 was reached. At this point, tetracycline (20 µg/mL) was added to stop de novo protein synthesis. Bacterial cultures were incubated under the same conditions and samples were taken every 15 min during 2 h to monitor SepL-FLAG, CesL-FLAG or untagged SepD protein levels by immunoblotting. Protein samples were normalized according to the OD_600_ value at each time point.

### 2.13. Structural Modeling of the SepL/SepD/CesL Complex

The models of the CesL and SepD proteins were generated using the I-TASSER server [76]. From the set of 5 possible models, the ones with the best C-score were chosen. For SepL modeling, residues 34-351 were threaded onto the solved structure of YopN32-277 from *Yersinia pestis* (PDB code 1XKP, chain A) [77] by specifying the use of YopN as a template. Once retrieved, each single protein model was separately fitted onto the crystal structure of the *Y. pestis* heterotrimeric complex (PDB code 1XKP) using the MatchMaker module implemented in UCSF Chimera [78] as follows: SepL was superimposed onto the structure of YopN, CesL onto the SycN structure and SepD onto the YscB structure. The orientation of the CesL protein model was manually adjusted so that the position of its C-terminus was consistent with the proposed SepD-binding region found in this work. The SepL/SepD/CesL heterotrimeric assembly was then refined with the GalaxyRefineComplex tool from GalaxyWEB server. GalaxyRefineComplex. Available online: http://galaxy.seoklab.org/ (last accessed on 12 December 2020) [79]. Molecular images were prepared using UCSF Chimera.

### 2.14. Coiled-Coil Prediction

Coiled-coil formation was calculated using COILS version 2.1 with a sliding window size of 21 residues. COILS. Available online: https://npsa-prabi.ibcp.fr/cgi-bin/npsa_automat.pl?page=/NPSA/npsa_lupas.html (last accessed on 27 December 2020) [80].

## 3. Results

### 3.1. CesL Differentially Regulates Translocator and Effector Protein Secretion

**Figure 1:**
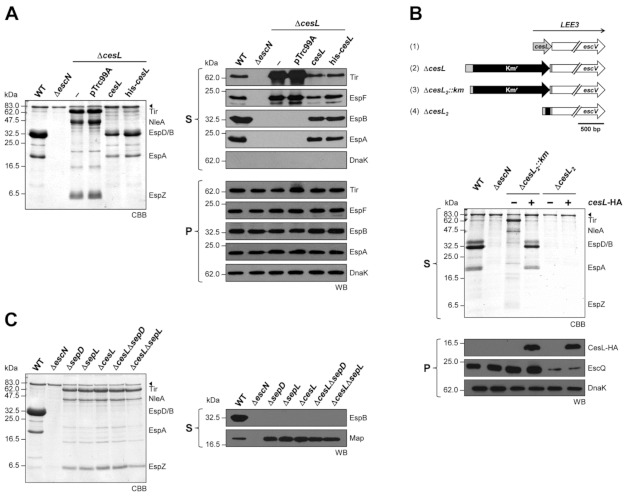
CesL protein is required for proper control of EPEC T3 secretion. (**A**) The EPEC wild-type strain (WT), Δ*escN* mutant strain, and Δ*cesL* mutant strain (-) carrying an empty vector (pTrc99A_FF4), plasmid pMTcL (*cesL*) producing untagged CesL or plasmid pMTHcL (*his-cesL*) producing His-CesL were grown under T3S inducing conditions and the secreted protein content was examined. Protein samples were resolved on SDS-PAGE and stained with Coomassie brilliant blue (CBB). Proteins corresponding to T3SS substrates or the autotransporter EspC (arrowhead) are indicated on the right. Secreted proteins (S) or whole-cell lysates (P) were analyzed by immunoblotting using antibodies against the indicated protein (WB). (**B**) In-frame deletion of the first gene in the *LEE3* operon results in a polar effect. Upper panel. Genetic organization of (1) wild-type *cesL* gene, (2) Δ*cesL* Km resistant mutant (3) equivalent CR *orf12* deletion mutant in EPEC but with the kanamycin cassette (Δ*cesL*_2_::km), (4) the latter mutant without the kanamycin cassette (Δ*cesL*_2_). Lower panel. Secreted proteins (S) and whole-cell lysates (P) of EPEC WT, the isogenic mutant Δ*escN*, and the *cesL* knock-out mutant with (Δ*cesL*_2_::km) or without (Δ*cesL*_2_) the kanamycin resistance cassette, and carrying (+) or not (-) pMATcL2HA plasmid expressing CesL-HA. Protein samples were analyzed by CBB-stained SDS-PAGE or immunoblotting with antibodies against the HA tag, the LEE3-encoded protein EscQ or the intracellular housekeeping chaperone DnaK. EspC is indicated with an arrowhead in CBB. (**C**) The EPEC Δ*cesL* mutant displays the same secretion phenotype as the Δ*sepD* and Δ*sepL* mutants. Protein secretion profile of EPEC WT, Δ*escN*, Δ*sepD*, Δ*sepL*, Δ*cesL* and the Δ*cesL*Δ*sepD* and Δ*cesL*Δ*sepL* double mutants was analyzed by CBB-stained SDS-PAGE (left panel) or by immunoblotting using anti-EspB and anti-Map antibodies (WB, right panel). EspC is indicated with an arrowhead in CBB.

The secretion phenotype of a CR *cesL* (*orf12*) mutant was reported to be a general defect in both translocator and effector protein secretion [54], and the same was observed for a *cesL* (*l0036*) mutant in EHEC [60]. In contrast, a recent study showed that an EPEC *cesL* mutant is unable to secrete the translocator EspA but hypersecretes the effector Tir [48]. These contrasting results regarding the *cesL* mutant phenotype among A/E pathogens are intriguing since the CesL proteins share more than 90% sequence identity. Here, we revisited the EPEC *cesL* mutant phenotype, demonstrating that CesL is indeed crucial for translocator protein secretion and negatively regulates effector protein secretion (Figure 1A). When compared to the wild-type strain, the secretion of EspA, EspB and EspD was completely abolished in the *cesL* mutant strain, while the effectors Tir, NleA, EspF, EspZ and Map were hypersecreted (Figure 1A CBB, panel S and Figure 1C, panel S). Furthermore, we showed that this phenotype is not due to an alteration in protein production in the *cesL* background (Figure 1A, panel P). Complementation of the *cesL* mutant with plasmids expressing CesL or an N-terminally His-tagged version of the protein (His-CesL), restored the secretion of translocators and diminished the hypersecretion of effectors to wild-type levels (Figure 1A, CBB and panel S). The *cesL* mutant phenotype was also complemented by a C-terminally double HA-tagged version of the protein (CesL-HA) (see Figure 5A), indicating that the *cesL* mutant is nonpolar and that neither the N- nor the C-terminal epitope tags interfere with CesL protein function. Therefore, CesL is involved in the regulation of the secretion hierarchy.

Based on these results, we investigated the difference between the previously reported *C. rodentium* and EHEC mutants versus the EPEC *cesL* mutant used in this study, which consists in the presence of a kanamycin cassette in the latter, while CR and EHEC were in frame deletions. Hence, we constructed the equivalent CR deletion mutant in EPEC (*cesL*_2_), as well as this same mutant with a kanamycin cassette (*cesL*_2_::km), and evaluated their phenotypes (Figure 1B). The results showed that the EPEC *cesL*_2_ deletion mutant exerted a polar effect on downstream genes essential for T3S which are encoded in the same operon, as shown by a considerably decreased level of the EscQ protein (Figure 1B, panel P, lanes Δ*cesL*_2_), thus impeding type III secretion and *in trans* mutant complementation (Figure 1B, panel S, lanes Δ*cesL*_2_). However, when the kanamycin resistance gene is present, the phenotype of the mutant was identical to that of the Δ*cesL* mutant and could be complemented *in trans* with plasmid-expressed CesL-HA (Figure 1B, panel S, lanes Δ*cesL*_2_::km). The *cesL* mutant used in this work is also represented in Figure 1B, top, (Δ*cesL*), in which sequences important for transcriptional regulation in the 5′ end of the gene were left intact [81].

In agreement with previous observations [48], we showed that the protein secretion profile of the *cesL* mutant is identical to the one reported for the *sepD* and *sepL* switch mutants (Figure 1C) [36,48,54]. In addition, we showed that the double mutants *cesLsepD* and *cesLsepL* exhibited the same phenotype as the single mutants (Figure 1C). These results are consistent with an earlier report showing that CesL associates with the SepD/SepL complex [59], and with our results of stable heterotrimeric complex formation (independently of which protein is tagged) (Figure S1), indicating that they act together in the regulation of ordered secretion.

### 3.2. Pairwise Protein–Protein Interactions of CesL with SepD and SepL

Based on a PSI-BLAST search as well as on secondary structure similarities, it was proposed that CesL is a homolog of SycN (a YopN gatekeeper chaperone), so it was classified as a class 1 chaperone for SepL, which in turn was shown to resemble an aberrant effector possessing a functional secretion signal [59]. However, a direct CesLSepL interaction has not been previously established. Hence, to characterize individual CesL interactions with SepD and SepL, we overproduced proteins in the same manner as for the heterotrimeric complex to perform co-purification assays under native conditions (see Figure S1). Nevertheless, upon protein induction of CesL with either SepL or SepD alone, CesL remained in the insoluble fraction; it was only soluble in the presence of both SepD and SepL (Figure S2). This result suggests that SepL and SepD interact with different domains of CesL, promoting its solubility.

**Figure 2:**
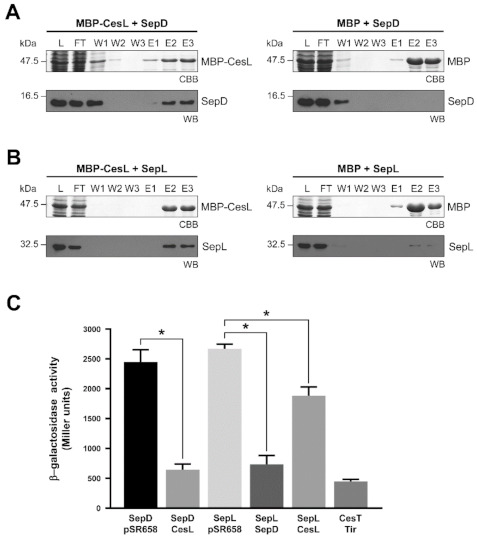
CesL associates independently with both SepD and SepL proteins. The MBP-CesL or MBP protein from a cleared cell lysate was immobilized on amylose resin and then a cleared lysate (L) containing either (**A**) untagged SepD or (**B**) untagged SepL was loaded onto the protein-coupled resin. Samples from the flow-through (FT), washes (W), and elutions (E) were collected and analyzed by CBB-stained SDS-PAGE and immunoblotting using anti-SepD or anti-SepL polyclonal antibodies. (**C**) Quantification of β-galactosidase activity of *E. coli* SU202 strain carrying plasmids pSR659 and pSR658 or derivatives thereof (expressing *sepD*, *sepL*, *cesL*, *tir* or *cesT* genes). The known SepD-SepL and CesT-Tir interactions were used as positive controls. Data represent the mean of three independent experiments in Miller units. Measurements were subjected to a 2-sided t-test to determine the statistical significance of the activity produced by the protein interactions compared to their respective negative control. * *p* < 0.005.

In order to improve the solubility of CesL for pull down assays, we generated a maltose binding protein (MBP) N-terminally tagged version of the protein (MBP-CesL). Co-purification assays showed that MBP-CesL was capable of retaining both untagged SepD (Figure 2A, left panel) and untagged SepL (Figure 2B, left panel), so that they co-eluted with MBP-CesL. MBP alone was used as a negative control (Figure 2A,B, right panels). It is worth noting that CesL shows an anomalous migration on SDS-PAGE.

These results were further confirmed using a bacterial two-hybrid system based on the *E. coli* LexA repressor (see Materials and Methods). In agreement with the abovementioned results, Figure 2C shows an interaction between CesL and both SepD and SepL, expressed as a statistically significant reduction in β-galactosidase activity compared to the respective protein tested against an empty vector. The previously known chaperone-effector (CesT-Tir) and the SepD-SepL protein-protein interactions [58,82,83], were used as positive controls. Taken together, these results showed that CesL individually interacts with SepL and SepD, both of which also interact with each other, forming the heterotrimeric complex that regulates hierarchical secretion.

**Figure 3:**
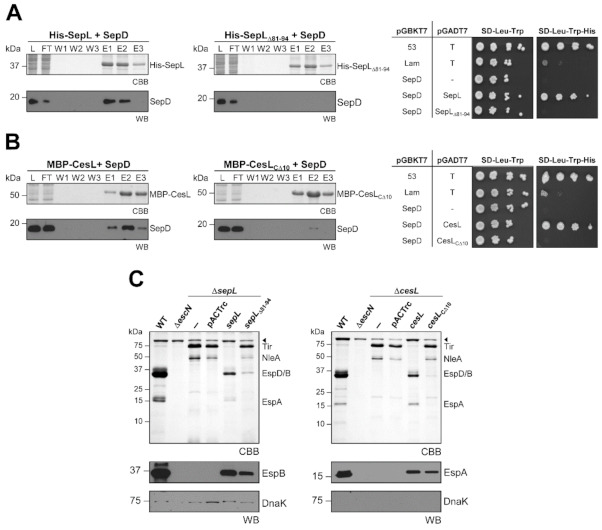
CesL and SepL interaction with SepD is required to regulate secretion hierarchy. (**A**) His-SepL or His-SepL_Δ81-94_ and (**B**) MBP-CesL or MBP-CesL_CΔ10_ recombinant proteins were immobilized on Ni-NTA or amylose resin, respectively, and then a cleared lysate (L) containing untagged SepD was loaded onto the protein-coupled resin. Samples from the flow-through (FT), washes (W) and elutions (E) were collected and analyzed by CBB-stained SDS-PAGE and immunoblotting using anti-SepD antibodies (left panels). *S. cerevisiae* PJ69-4a/α strain carrying plasmids pGADT7 and pGBKT7 or their indicated derivatives was serially diluted and spotted onto SD-Leu-Trp medium as a growth control, and onto SD-Leu-Trp-His medium to test for protein interactions. The plasmid pairs pGADT7-T/pGBKT7-53 and pGADT7-T/pGBKT7-Lam were used as positive and negative controls, respectively. The plasmid pair pGBKT7-SepD/pGADT7-T was used as a SepD self-activation control (right panels). **(C)** Secreted proteins from EPEC wild-type strain (WT), Δ*escN* strain*,* and Δ*sepL* (-) or Δ*cesL* (-) strains harboring an empty vector (pACTrc), plasmid pMATpL expressing SepL, plasmid pMATpL_Δ81-94_ expressing SepL_Δ81-94_, plasmid pMATcL expressing CesL or plasmid pMATcL_CΔ10_ expressing CesL_CΔ10_, were resolved on CBB-stained SDS-PAGE. Immunoblotting of EspB, EspA and DnaK proteins is shown (WB).

Furthermore, to dissect interaction domains between these proteins, we constructed truncated versions and analyzed their ability to interact with each other. The CesL interaction site on SepL maps to the amino terminal region within amino acids 30-75, since a SepL truncated version lacking the first 30 amino acids (SepL_Δ30_) is able to form the heterotrimeric complex, while a SepL version lacking the first 75 amino acids (SepL_Δ75_) forms a complex in which the interaction with SepD is not significantly affected but the one with CesL is severely diminished (Figure S3). This result further indicates that the SepD interaction site on SepL is beyond amino acid 75. Analysis of the SepL protein sequence revealed a predicted coiled-coil motif between amino acids 71-94 (see Materials and Methods), suggesting that such a motif may be important for the SepL–SepD interaction. Hence, we constructed a SepL deletion version lacking amino acids 81-94 (SepL_Δ81-94_) and assessed its interaction with SepD by co-purification and yeast two hybrid assays. The results showed that the protein interaction was abolished in both assays (Figure 3A), indicating that this SepL region is important for SepD binding. Moreover, it has been reported that a coiled-coil motif at the C-terminal region of the *Salmonella* SPI-2 encoded SsaM protein (a CesL homologue), is important for binding SpiC (a SepD homologue) [56]. Thus, although the CesL protein nowhere contains a predicted coiled-coil, a deletion version of this protein lacking the last 10 amino acids was constructed (CesL_CΔ10_), and its interaction with SepD was found to be considerably affected (Figure 3B). Considering these results, we evaluated the secretion phenotype of both mutant versions. The expression of either SepL_Δ81-94_ or CesL_CΔ10_ from a low-copy number plasmid in the Δ*sepL* or Δ*cesL* background, respectively, did not reduce effector hypersecretion, but low level of translocators were found to be secreted, in contrast to complementation with full-length SepL or CesL that secretes similar levels of effectors and translocators to the wild-type strain (Figure 3C). These results suggest that formation of the heterotrimeric complex is important for proper hierarchical secretion and highlights the relevance of the interactions between CesL and SepD, and SepL and SepD, mainly to inhibit effector secretion.

While the structure of almost the entire SepL protein (amino acids 80-350) has been solved [84], there is no structural information for neither SepD nor CesL proteins. Here, we built a model of the tertiary structure of the SepL/SepD/CesL heterotrimeric complex based on the crystal structure of the YopN-TyeA/YscB/SycN complex of *Y. pestis* (PDB ID:1XKP). The three dimensional structures of CesL and SepD were modeled using the I-TASSER prediction server [76]. Remarkably, the top templates that the algorithm used to generate the CesL and SepD structural models were SycN and other T3SS-related chaperones, respectively. As the N-terminal domain of YopN provides most of the contacts for its cognate partners, and such region of SepL has not been solved yet, we decided to model the SepL structure by guiding the algorithm using the YopN structure as template (see Materials and Methods). The predicted structures were fitted onto their respective counterpart on the YopN-TyeA/YscB/SycN complex, depicting the SepL/SepD/CesL heterotrimeric complex (Figure S4). The model suggests a larger molecular interface between CesL and SepD than the one between CesL and SepL, where only a very discrete portion of the SepL N-terminus is mediating the interaction.

### 3.3. Stability of Both CesL and SepL Is Affected in the Absence of Each Other

**Figure 4:**
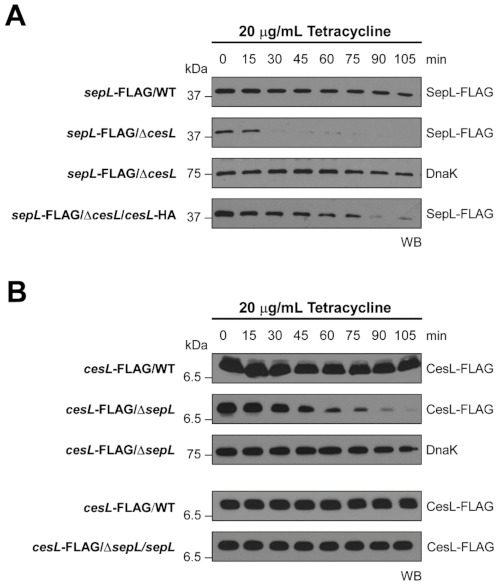
Interdependent stability of SepL and CesL proteins. (**A**) Immunoblotting showing the protein levels of chromosomally encoded SepL-FLAG expressed in wild-type EPEC (WT), Δ*cesL* mutant, and the Δ*cesL* strain carrying plasmid pMATcL2HA producing CesL-HA. (**B**) Immunoblotting showing the protein levels of chromosomally encoded CesL-FLAG in wild-type EPEC (WT), Δ*sepL* mutant, and the Δ*sepL* strain carrying plasmid pMATpL expressing untagged SepL. After halting de novo protein synthesis by addition of tetracycline, whole cell samples were collected every 15 min during 105 min. DnaK was used as a protein loading control. Samples were normalized according to the culture OD_600_ at the time of collection.

As abovementioned, CesL was proposed to be a chaperone for SepL [59]. To evaluate if CesL enhances the stability of SepL we performed protein stability assays in the presence of tetracycline to inhibit de novo protein synthesis. As shown in Figure 4A, the stability of functional chromosomally produced SepL-FLAG is negatively affected in the *cesL* mutant background, and this effect could be partially complemented when introducing a plasmid encoding CesL-HA. Besides, we also analyzed the stability of functional chromosomally produced CesL-FLAG. The result was unexpected for a predicted class 1 chaperone, as CesL stability was reduced in the *sepL* mutant background (Figure 4B). The CesL-FLAG stability defect could be complemented to wild-type levels when introducing the *sepL* gene *in trans* (Figure 4B). We also observed that the stability of SepD decreased in the absence of SepL and CesL, and that the stability of SepL and CesL is affected in a *sepD* mutant background (Figure S5). These results indicate that the stability of the three switch components is affected in the absence of each of their interacting partners, suggesting they function together as a protein complex. In this study we propose a model for the translocon to effector switching mechanism in EPEC, which depicts for the first time the existence of a heterotrimeric complex (see Discussion).

### 3.4. CesL Is Not Secreted and Localizes to the Membrane Independently of SepD and SepL

**Figure 5:**
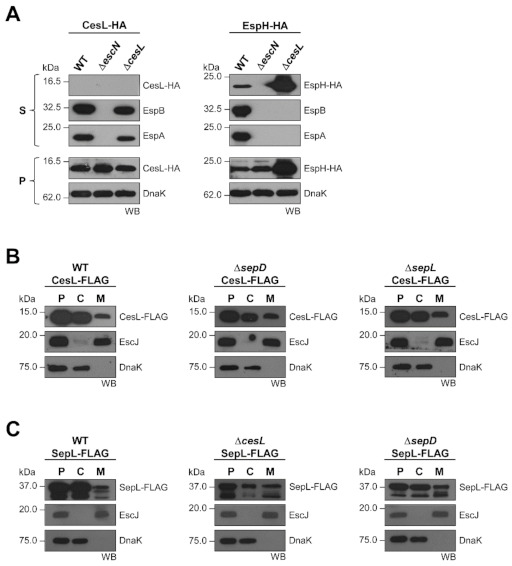
CesL and SepL associate with the membrane independently of their molecular switch interacting partners. (**A**) Secreted proteins (S) and whole-cell lysates (P) of wild-type EPEC (WT), Δ*escN* and Δ*cesL* strains harboring plasmid pMHcL producing CesL-HA (left panel), or plasmid pJHeH producing EspH-HA (right panel), were subjected to SDS-PAGE and immunoblotting with an anti-HA antibody. The presence of EspB and EspA in the supernatant and that of DnaK in the bacterial pellet was also analyzed by immunoblotting. (**B**,**C**) Wild-type EPEC (WT), Δ*sepD* and Δ*sepL* strains expressing chromosomally encoded (**B**) CesL-FLAG or (**C**) SepL-FLAG. EPEC strains were grown under T3S-inducing conditions and fractionated into cytoplasmic (C) and membrane (M) fractions. Whole-cell lysates (P) were loaded as a control. EscJ and DnaK were used as cytoplasmic and membrane protein controls, respectively. Equal amounts of each fraction were probed using antibodies against the FLAG tag, EscJ, or DnaK.

It is well known that various gatekeeper proteins are secreted in order to allow timely effector translocation [39,43,44]. Likewise, some proteins that form the gatekeeper complex have been reported to be secreted or translocated [44,47]. Thus, we evaluated whether CesL could be a T3SS substrate. The secretion profile of an HA C-terminally tagged version of CesL (CesL-HA) showed that this protein was not recovered in the supernatant fraction even though the translocators EspA and EspB were secreted (Figure 5A, left panel). In contrast, the EspH-HA effector was secreted in the wild-type strain and hypersecreted in the *cesL* mutant background, but not in the ATPase mutant used as a negative control (Figure 5A, right panel). The DnaK cytoplasmic chaperone was used as a loading control for whole cells.

In order to determine the localization of CesL, bacteria were fractionated into cytoplasmic and membrane fractions, and the protein was identified using antibodies against the C-terminal FLAG tag, as detailed in Materials and Methods. In wild-type EPEC, CesL was detected in both the cytoplasmic and membrane fractions (Figure 5B, left panel), which is consistent with the location previously reported for SepL and SepD [36]. The inner membrane ring protein EscJ was used as a membrane fraction control and DnaK as a cytoplasmic fraction control. We next examined whether the localization of CesL was affected in the absence of the other components of the gatekeeper complex. As seen in Figure 5B (middle and right panels), the localization of CesL-HA was not altered in the *sepD* and *sepL* mutants. This result suggests that CesL is intrinsically targeted to the inner membrane.

Additionally, we evaluated if the membrane localization of SepL was dependent on CesL and SepD. Chromosomally produced FLAG tagged SepL (SepL-FLAG) was localized in both the cytoplasmic and membrane fractions independently of the presence of CesL and SepD (Figure 5C). In agreement, the same result was obtained when using untagged SepL expressed from a low-copy number plasmid (Figure S6).

### 3.5. CesL Interacts with Both Components of the Export Gate

**Figure 6:**
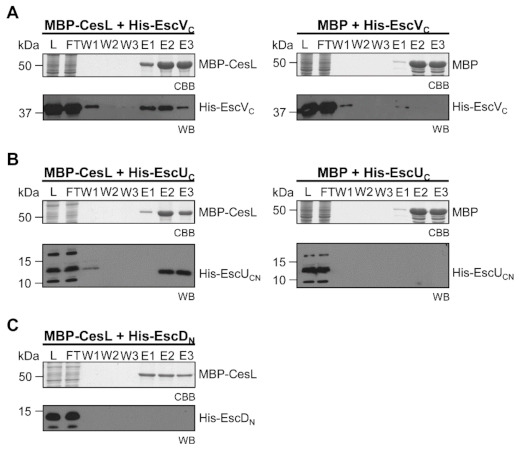
CesL interacts with the export gate components EscV and EscU. MBP-CesL (left panels) or MBP (right panels) was bound to a packed amylose resin and a cleared lysate (L) containing the His-tagged cytoplasmic domain of (**A**) EscV (EscV_C_) or (**B**) EscU (EscU_C_) and the mixture was loaded into the column. Samples from the flow-through (FT), washes (W) and elutions (E) were collected and analyzed by CBB-stained SDS-PAGE and immunoblotting (WB) using anti-EscV_C_ or anti-EscU_C_ polyclonal antibodies. (**C**) The His-tagged cytoplasmic domain of EscD (EscD_N_) did not copurify with MBP-CesL and was used as a negative control.

The cytoplasmic C-terminal domain of two of the membrane components of the export apparatus (EscU_C_ and EscV_C_), forms the export gate through which substrates are secreted [5]. We and others have previously demonstrated that the gatekeeper SepL interacts with the soluble domain of EscV and that this interaction is important for a proper secretion hierarchy [48,49]. Likewise, here we show that CesL interacts with EscV_C_ (Figure 6A), suggesting that it is the SepL/SepD/CesL complex the one modulating the conformational changes in EscV that lead to differential substrate recognition. Remarkably, the CesL protein also interacted with EscU_C_ (Figure 6B), which is involved in the first specificity switching event, from early to intermediate and late substrates [35,85,86]. MBP alone was used as a negative control (Figure 6A,B, right panels). Moreover, we tested the MBP-CesL interaction with another IM protein, EscD, which forms one of the basal body inner membrane rings. The result showed that the soluble N-terminal domain of the EscD protein (EscD_N_) did not interact with MBP-CesL, indicating the non-existence of an MBP-CesL unspecific interaction (Figure 6C).

## 4. Discussion

The T3SS is a recurrent theme in bacterial pathogenesis. The proper assembly and functioning of this sophisticated nanomachine depends on the orchestrated secretion of its component proteins [33,87]. This process is regulated by two molecular specificity switches, which guarantee that bacterial effectors are delivered directly into the host cell [5]. Although recent advances have markedly increased our understanding of the function of these two regulatory complexes, the precise molecular mechanisms preventing a futile secretion of effectors are largely unknown. In EPEC, the SepL/SepD complex has been found to have a major role in the regulation of substrate specificity switching [36,48]. Our data shows that the CesL component of the gatekeeper complex has a direct role in fine-tuning the orderly secretion of T3 substrates. We propose a model in which CesL facilitates a crosstalk between the two molecular switches.

Contrasting phenotypes have been reported for *cesL* deletion mutants in CR, EHEC and EPEC [48,54,60]. Hence, as a first step to clarify the function of CesL, we constructed a set of *cesL* deletion strains in EPEC with and without a kanamycin resistance cassette. Consistent with the phenotype reported by Portaliou et al. [48], we have shown that CesL is essential for translocator secretion and negatively regulates effector secretion (Figure 1A), in an identical manner as *sepL* and *sepD* mutants (Figure 1C). Further, we showed that the different phenotypes are attributed to the presence/absence of the km resistance cassette. The in frame deletion of *cesL* in EPEC (Δ*cesL*_2_) had a polar effect on the expression of downstream genes in the *LEE3* operon *cesL*, *escV*, *escN*, *escO, escP*, *escQ* and *espH* (all of which are essential for T3S except for *espH*), and thus protein secretion was abolished (Figure 1B). However, this effect was overcome by the insertion of the km cassette, which probably drives the transcription and subsequent translation of downstream genes. This result is consistent with what has been observed in EHEC, where translation of *l0036* (*cesL*) is needed for the synthesis of downstream gene products [60]. However, our results indicate that it is not the translation of *cesL* per se, but that of any other gene, which presumably protects the *LEE3* transcript from being degraded. Our proposal is in agreement with recent observations for the *LEE5* operon, where translation of *tir*, the first gene of the operon, is needed to prevent degradation of the *LEE5* transcript [88]. Overall, our results confirm a role of CesL in the regulation of ordered secretion.

The EPEC SepL/SepD/CesL ternary complex resembles the *Salmonella* SsaL/SpiC/SsaM, *Yersinia* YopN-TyeA/YscB/SycN, *Pseudomonas* PopN-Pcr1/PscB/Pcr2, *Chlamydia* CopN/Scc4/Scc1 and *Edwarsiella* EsaL/EsaB/EsaM regulatory complexes needed for the organized secretion of T3 substrates in homologous systems [42,44,46,59,77,89]. It has been reported that the N-terminal region of some gatekeepers such as YopN, PopN and CopN interacts with heterodimeric chaperone complexes (YscB/SycN, PscB/Pcr2 and Scc4/Scc1, respectively), which stabilize and target the gatekeeper to the export apparatus for its secretion [44,55,77]. However, unlike these previous reports, we showed that CesL and SepD can bind to SepL independently (Figure 2), suggesting that a preformed SepD/CesL chaperone complex is not required for binding to the gatekeeper. These pairwise interactions are in agreement with those reported for the EsaL/EsaB/EsaM complex in *E. tarda*, where EsaB (SepD homologue) and EsaM (CesL homologue) can individually interact with EsaL (SepL homologue). Additionally, in accordance to what we observed, the EsaB and EsaM proteins can interact between them independently of EsaL [42].

Coiled-coil motifs have been found to mediate protein-protein interactions and their importance in T3S systems have been previously reported [90]. SepL possesses a predicted coiled-coil motif at its N-terminal domain, and remarkably, several SepL homologues share this structural characteristic in the same region. Here, we demonstrate that the integrity of this motif is crucial for SepD binding (Figure 3A). It is noteworthy that the atomic structure of this region of SepL remains unsolved [84]. Likewise, we found that the C-terminal region of CesL is also critical for SepD interaction (Figure 3B). Functional analysis further confirmed the importance of these interfaces for secretion regulation (Figure 3C). Remarkably, both truncated versions that abrogate the interaction with SepD were unable to prevent effector hypersecretion in complementation assays. On the other hand, the secretion of translocators was partially restored. These results suggest that the main role of SepD in hierarchy regulation is to stop premature effector secretion, and that a proper heterotrimeric complex must be formed to achieve this function.

Furthermore, our results suggest that the interaction region between CesL and SepL maps within SepL amino acids 30 to 75 (Figure S3). The interacting domains at the N-terminus of SepL are supported by our in silico modeling of the gatekeeper complex, in which this region adopts an extended conformation that binds both CesL and SepD (Figure S4). Further structural studies of the entire heterotrimeric complex are needed to define the precise arrangement of its components.

CesL and SepD were previously proposed to function as chaperones [59]. In agreement with this role, the stability of SepL was severely affected in the absence of these proteins. However, unexpectedly, the stability of CesL and SepD was also compromised in a *sepL* mutant background. Therefore, formation of the trimeric complex is important for protein stability. Albeit that CesL is able to interact in vitro with SepL and SepD in a pairwise manner, our study demonstrates that the formation of the heterotrimeric complex is crucial for stability of these proteins within bacterial cells (Figure 4 and Figure S5).

Several gatekeeper proteins are known to be secreted in a T3SS-dependent manner; however, we showed that CesL is not secreted into the culture supernatant (Figure 5A). Nevertheless, it was recently reported that the *Vibrio parahaemolyticus* gatekeeper proteins VgpA and VgpB are translocated into host cells [47]. Since these proteins are not secreted in vitro, further studies will be needed to explore an additional extracellular role of EPEC switch proteins during the infection process. Ours and other previous studies have shown that SepL and its gatekeeper binding partners exist in both a soluble and a membrane-associated pool [36,49,57,91]. In this work we showed that CesL and SepL displayed the same localization pattern independently of other components of the gatekeeper complex (Figure 5B,C and Figure S6). These results are consistent with a previous report showing that SepL localization is not affected in the absence of SepD [48], but are in contrast with data showing that the membrane localization of untagged SepL or a SepL-eGFP version is disrupted in the absence of CesL [48] or of SepD [91], respectively. These discrepancies could be due to methodological differences in the fractionation procedure, or to the presence of a large tag such as the GFP protein.

The T3S inner membrane proteins EscV and EscU are crucial components of the EPEC injectisome, and their large cytoplasmic domains reside at the entry of the export gate, where they have direct access to T3 substrates right before entering the secretion pathway. Here, we found that in addition to SepL, CesL is able to interact with EscV. Moreover, we report the formation of a novel protein complex composed by CesL and EscU (Figure 6). The functional relevance of these CesL complexes remains unclear, although we propose a model where they might be coupling the first and second molecular switches (see below).

Taken together, our data suggest that CesL does not act as a canonical chaperone, but rather plays an active role in the regulation of the secretion hierarchy.

**Figure 7:**
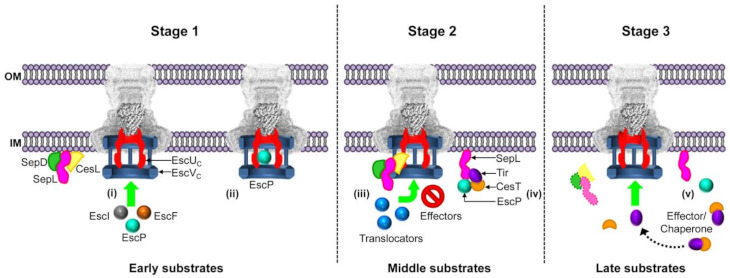
Model for regulation of type III secretion hierarchy. During T3SS assembly, two molecular switches ensure that the different substrates are secreted in a hierarchical order. **Stage 1.** (**i**) Early substrates are secreted. At this time, the molecular switch proteins SepL, SepD and CesL (in complex or independently), are associated with the membrane and likely located near the export apparatus. (**ii**) When the needle reaches its predetermined length, EscP interacts with EscU_C_, promoting a conformational change that flips the first substrate specificity switch, thereby halting early substrate secretion and allowing (together with the SepL/SepD/CesL complex) middle substrate recognition (see next stage). **Stage 2.** (**iii**) The first specificity switching event is transmitted to the EscV export gate component via interactions of the SepL/SepD/CesL heterotrimeric complex with both EscU and EscV. This communication enables middle substrates to be recognized and secreted by the EscV docking platform. (**iv**) At the same time, EscP bound to SepL sequesters the chaperone-effector CesT/Tir complex. **Stage 3.** (**v**) Once the translocation pore has been inserted into the host cell membrane, an activation signal is transmitted to the export apparatus, which results in switch complex dissociation, permitting late substrates binding to EscV and effector translocation. In addition, it has been shown that a drop in calcium concentration dissociates the SepL–EscP interaction, releasing the CesT/Tir complex for docking at EscV.

Taking into account the data presented here and results from previous studies, we propose a model for regulation of EPEC type III secretion as shown in Figure 7. (i) EscU_C_ (SctU_C_) is autocleaved into two subdomains, EscU_CN_ and EscU_CC_ that remain tightly associated [85,86,92]. In the first assembly stage, autocleaved EscU and the ruler protein EscP modulate the secretion of the early substrates EscI and EscF, necessary for the assembly of the inner rod and needle. During formation of these structures EscP is also occasionally secreted [35]. (ii) When the needle reaches its proper length, EscP productively interacts with EscU to stop secretion of early substrates and to streamline the substrate specificity switching to middle substrates, allowing recognition of translocators and positively influencing the SctW gatekeeper interaction with the SctV export gate component, as reported for *Salmonella* SPI-2 [35,50]. (iii) In this same regard, we suggest that the CesL-EscU interaction demonstrated in this work could play a role in promoting the SepL-EscV interaction, thereby communicating that the first substrate switching event has occurred. In this second stage, translocators are secreted while, simultaneously, the premature secretion of effectors is blocked. This is regulated by the SepL/SepD/CesL molecular switch in complex with EscV, which works as a docking platform with high affinity for translocator-chaperone complexes and which inhibits binding of effector-chaperone complexes [48,49]. (iv) It has been reported that SctU is present in a stoichiometry of one subunit within the export apparatus [22,93], thus it is possible that the interaction with one EscP molecule could flip the specificity switch. The remnant EscP protein could be located at the inner membrane in complex with SepL, sequestering the CesT/Tir complex, since interactions between these proteins have been observed, and EscP has been reported to be associated with the membrane [35,61,91]. (v) Once the EspA filament has been assembled and the translocation pore is formed in the host cell membrane, the cell contact signal is transmitted to the export apparatus through conformational changes in the filament/needle [37,38,39,40]. Consequently, the SepL/SepD switch proteins (in complex with CesL shown here to interact with EscVc), disengage from EscV allowing effector secretion. The EscVc platform thereby changes its conformation to bind effector-chaperone complexes with high affinity [48]. In addition, the formation of a SepL-EscP complex was reported to participate in regulation of effector secretion [61]. It has been suggested that host cell contact produces a drop in calcium concentration at the base of the injectisome [36], which was shown to disrupt the SepL–EscP interaction [61], which in turn releases the CesT-Tir complex to initiate effector translocation [35,61].

Our working model regarding the functionality of the newly described EscU-CesL interaction is supported by recent experimental evidence from the *Salmonella* SPI-2 T3SS, where in the absence of SsaP (SctP), or in the presence of a non-cleaved form of SsaU (SctU), the binding of SsaV (SctV) with the gatekeeper SsaL (SctW) is diminished [50]. Moreover, in the flagellar system it has been shown that the interaction of FliK (SctP) with the cleaved form of FlhB_C_ (SctU_C_), ends the secretion of hook type substrates (early substrates in the injectisome) and promotes structural changes in the cytoplasmic domain of FlhA (SctV). The direct interaction of FlhB_C_ with FlhA_C_ enables the latter to adopt an open conformation that recognizes the next substrate category, namely, filament type substrates (middle substrates in the injectisome) [94,95,96]. Overall, these data indicate the existence of an interconnection between both molecular switches. Moreover, our results showed an additional direct role of CesL in the secretion process and contribute to increase our understanding of the molecular switches and how they are related to each other in order to define the secretion hierarchy.

In addition to the main function of the gatekeeper complex in switching substrate specificity from translocators to effectors, current knowledge indicates that the gatekeeper SctW also regulates the secretion of early substrates by promoting the docking of chaperone-substrate complexes to the ATPase SctN [97], highlighting the relevance of this protein complex in the T3SS.

Therefore, the knowledge gained on the functioning of the gatekeeper complex could have implications for its use as a therapeutic target, or as a secretion regulator of a biotechnological vehicle to inject modified proteins. Indeed, the T3SS of EPEC has already been genetically engineered to translocate proteins of interest into mammalian cells [98].

## Figures and Tables

**Table 1:** Strains and plasmids used in this study.

Strain or Plasmid	Description ^1^	Reference or Source
*E. coli*		
TOP10	Strain used for cloning; Sm^r^	Invitrogen
XL1-Blue	Strain used for plasmid propagation and DNA purification; Tc^r^	Stratagene
BL21 (DE3) pLysS	Strain used for expression of pET19b based plasmids; Cm^r^	Novagen
SU202	Reporter strain for LexA-based two-hybrid assays; Cm^r^ Km^r^	[62]
EPEC E2348/69	Wild-type EPEC strain O127:H6; Sm^r^	[63]
Δ*escN*	E2348/69 carrying an in-frame deletion of *escN*; Sm^r^	[64]
Δ*cesL*	E2348/69 *cesL* deletion mutant. Codons 49 to 106 were replaced by the *aphT* cassette; Sm^r^ Km^r^	This study
Δ*cesL*_2_::km	E2348/69 *cesL* deletion mutant. Codons 20 to 106 were replaced by the *aphT* cassette; Sm^r^ Km^r^	This study
Δ*cesL*_2_	Derived from Δ*cesL*_2_::km, the kanamycin cassette was removed; Sm^r^	This study
Δ*sepD*	E2348/69 carrying an in-frame deletion of *sepD*; Sm^r^	Gift of the Puente JL Lab
Δ*sepL*	E2348/69 carrying an in-frame deletion of *sepL*; *S*m^r^	Gift of the Puente JL Lab
Δ*cesL* Δ*sepD*	E2348/69 *cesL* and *sepD* double deletion mutant; Sm^r^ Km^r^	This study
Δ*cesL* Δ*sepL*	E2348/69 *cesL* and *sepL* double deletion mutant; Sm^r^ Km^r^	This study
*sepL*-FLAG	E2348/69 expressing 3×FLAG-tagged SepL; Sm^r^ Km^r^	[49]
*sepL*-FLAG Δ*cesL*	E2348/69 expressing 3×FLAG-tagged SepL and carrying a deletion of *cesL*; Sm^r^ Km^r^	This study
*sepL*-FLAG Δ*sepD*	E2348/69 expressing 3×FLAG-tagged SepL and carrying an in-frame deletion of *sepD*; Sm^r^ Km^r^	This study
*cesL*-FLAG	E2348/69 expressing 3×FLAG-tagged *cesL*; Sm^r^ Km^r^	This study
*cesL*-FLAG Δ*sepL*	E2348/69 expressing 3×FLAG-tagged CesL and carrying an in-frame deletion of *sepL*; Sm^r^ Km^r^	This study
*cesL*-FLAG Δ*sepD*	E2348/69 expressing 3×FLAG-tagged CesL and carrying an in-frame deletion of *sepD*; Sm^r^ Km^r^	This study
*Salmonella*		
SJW1368	Strain used for expression of pTrc99A-based plasmids; flagellar master operon mutant, Δ(*cheW*-*flhD*)	[65]
JR501	Strain used to convert *E. coli* derived plasmids to *Salmonella* compatibility	[66]
*Saccharomyces cerevisiae*		
PJ69-4a/*α*	*MATa/α trp1*-*901 leu2*-*3*, *112 ura3*-*52 his3*-*200 gal4*Δ *gal80*Δ *LYS2*::*GAL1*-*HIS3 GAL2*-*ADE2 met2*::*GAL7*-*lacZ*	[67]
pKD4	Template plasmid for amplification of the kanamycin resistance cassette	[68]
pKD46	λ-Red recombinase system plasmid with an inducible *araB* promoter; Ap^r^	[68]
pSUB11	Template plasmid for amplification of the 3x-FLAG epitope and kanamycin resistant cassette; Ap^r^ Km^r^	[69]
pFLP2	Plasmid used for expression of the Flp recombinase; Ap^r^	[70]
pTrc99A	Expression vector with an inducible *trc* promoter; Ap^r^	Amersham-Pharmacia
pTrc99A_FF4	Modified pTrc99A expression vector with an inducible *trc* promoter; Ap^r^	[71]
pMTcL	*cesL* cloned into pTrc99A_FF4 (NdeI- BamHI)	This study
pMTHcL	*his-cesL* cloned into pTrc99A (NcoI-BamHI)	This study
pATpD	*sepD* cloned into pTrc99A_FF4	[49]
pMTpL	*sepL* gene cloned into pTrc99A_FF4	[49]
pMTBISpDcL	*sepD* and *his-cesL* cloned into pTrc99A_FF4	[49]
pMTBISpDpL	*sepD* and *his-sepL* cloned into pTrc99A_FF4	This study
pMTBISpLcL	*sepL* and *his-cesL* cloned into pTrc99A_FF4	This study
pMTBISpLpD	*sepL* and *his-sepD* cloned into pTrc99A_FF4	This study
pMTBISpDpL_Δ75_	*sepD* and *his-sepL* lacking codons 1 to 225 cloned into pTrc99A_FF4	This study
pET19b	Plasmid used for expression of His tagged proteins under the control of an inducible T7 promoter, Ap^r^	Novagen
pAEpD	*sepD* cloned into pET19b (NdeI-BamHI)	This study
pMEcL	*cesL* cloned into pET19b	[49]
pMEpL	*sepL* cloned into pET19b (NdeI-BamHI)	This study
pMEpL_Δ75_	*sepL* lacking codons 1 to 225 cloned into pET19b (NdeI-BamHI)	This study
pMEpL_Δ81-94_	*sepL* lacking codons 240 to 282 cloned into pET19b (NdeI and BamHI)	This study
pKEeD_N_	*escD* codons 1 to 120 cloned into pET19b	[49]
pKEeV_C_	*escV* codons 335 to 675 cloned into pET19b	[49]
pJEeU_C_	*escU* codons 215 to 345 cloned into pET19b	[35]
pACTrc	pACYC184 expression vector derivative; pTrc promoter, p15A origin of replication, *lacIq*; Cm^r^	[72]
pMATpD	*sepD* gene cloned into pACTrc (NdeI-BamHI)	This study
pMATpL	*sepL* gene cloned into pACTrc	[49]
pMATpL_Δ30_	*sepL* lacking codons 1 to 90 cloned into pACTrc (NdeI-BamHI)	This study
pMATpL_Δ81-94_	*sepL* lacking codons 240 to 282 cloned into pACTrc (NdeI-BamHI)	This study
pMATcL	*cesL* cloned into pACTrc (NdeI- BamHI)	This study
pMATcL2HA	*cesL*-2HA cloned into pACTrc (HindIII-BamHI)	This study
pMATcL_CΔ10_	*cesL* lacking codons 107-117 cloned into pACTrc (NdeI-BamHI)	This study
pMAL-c2X	Plasmid used for expression of MBP-tagged proteins under the control of the *tac* promoter; Ap^r^	New England Biolabs
pMLcL	*cesL* gene cloned into pMAL-c2X (BamHI-HindIII)	This study
pMLcL_CΔ10_	*cesL* lacking codons 107-117 cloned into pMAL-c2X (BamHI-PstI)	This study
pTOPO-2HA	pCR2.1-TOPO derivative carrying *C. rodentium espG* (HindIII-XhoI) tagged with 2-HA epitopes; Km^r^ Ap^r^	[36]
pMHcL	*cesL* with its native RBS cloned into pTOPO-2HA (HindIII-XhoI)	This study
pJHeH	*espH* with its native RBS cloned into pTOPO-2HA (HindIII-XhoI)	[35]
pGBKT7	Y2H vector containing GAL4 DNA binding domain; *TRP1* nutritional marker	Clontech
pOGBpD	*sepD* gene cloned into pGBKT7 (NdeI-BamHI)	This study
pGBKT7-53	pGBKT7 encoding a fusion of the GAL4 DNA binding domain with murine p53	Clontech
pGBKT7-Lam	pGBKT7 encoding a fusion of the GAL4 DNA binding domain with human lamin C	Clontech
pGADT7	Y2H vector containing GAL4 activation domain; *LEU2* nutritional marker	Clontech
pMGADpL	*sepL* cloned into pGADT7 (NdeI-BamHI)	This study
pOGADpL_Δ81-94_	*sepL* lacking codons 81-94 cloned into pGADT7 (NdeI-BamHI)	This study
pMGADcL	*cesL* gene cloned into pGADT7 (NdeI-BamHI)	This study
pMGADcL_CΔ10_	*cesL* lacking codons 107-117 cloned into pGADT7 (NdeI-BamHI)	This study
pGADT7-T	pGADT7 encoding a fusion of the GAL4 activation domain with the simian virus 40 (SV40) large T antigen	Clontech
pSR658	Encodes LexA DNA binding domain (WT). ColE1 origin of replication; Tc^r^	Gift of the Puente JL Lab
pMR58cL	*cesL* cloned into pSR658 (XhoI-KpnI)	This study
pMR58pD	*sepD* cloned into pSR658 (XhoI-KpnI)	This study
pMR58tir	*tir* cloned into pSR658 (XhoI-KpnI)	This study
pSR659	Encodes LexA DNA binding domain (Mut). p15A origin of replication; Ap^r^	Gift of the Puente JL Lab
pMR59cT	*cesT* cloned into pSR659 (KpnI-BamHI)	This study
pMR59pD	*sepD* cloned into pSR659 (XhoI-KpnI)	This study
pMR59pL	*sepL* cloned into pSR659 (BamHI-XhoI)	This study

^1^ Sm: streptomycin; Km: kanamycin; Cm: chloramphenicol; Tc: tetracycline.

**Table 2:** Oligonucleotides used in this study.

Oligonucleotide	Sequence 5′ to 3′
delcesLSRS_Fw	AACCGTGTTGAAATTGATTTTAATGGGTTTTCTTTTTTTATTGAAATAATTGATAATAATGTGTAGGCTGGAGCTGCTTCGAAGTTCCTATA
delcesL_Rv	ATTTAAGAGTTTATTCATGATGTCATCCTGCGAACGCGCTCAATAATCTGAATATCCTCCTTAGTTCCTA
delcesL_Fw	TTTTAGTTAAAAGAAATGTTGAAGAGTTTTTAAGATTGTTGGGAAATGATGTGTAGGCTGGAGCTGCTTC
sepL-3FLAG_Fw	ATACATTATTAATGATTGGTAAAGTGATAGATTATAAGGAGGATGTTATGGACTACAAAGACCATGACGG
sepL-3FLAG_Rv	CCTCTTCATAATCTTTCTTAGCATGACAAAAACTATAAAAAAAAACAATAATGAATATCCTCCTTAGTTC
cesL-3FLAG_Fw	GAATACTTTTCAACAGCATGTGCAGATTATTGAGCGCGTTCGCAGGATGACATCAGACTACAAAGACCATGACGG
cesL-3FLAG_Rv	AAGATCGTGATATGACTCTGCTTTTTTAAATATATTTAAGAGTTTATTCATATGAATATCCTCCTTAGTTC
sepLNdeI_Fw	AGTTTCATATGGCTAATGGTATTG
sepLNdeIΔ30_Fw	GCAATTACATATGCAAAAAAATTC
sepLNdeΔ75_Fw	GAATTTAATCATATGCCCGCATCT
sepLBamHI_Rv	CTATAAAAAAAAGGATCCTCACAT
sepLΔ81-94-A_Rv	ACCGATAGTGATAAAATAAAAGAA
sepLΔ81-94-B_Fw	ATCACTATCGGTTGTCGTGCCTTC
cesLBamHI_Fw	AGCCTGGGATCCAATCTTTTAGT
cesLHindIII_Rv	ATTTAAAAGCTTATTCATGATGTC
cesLMalPstI_Rv	ACGCGCTGCAGTTACTGCACATGC
cesLNdeI_Fw	AGAGCCTGCATATGAATCTTTTAG
cesLCΔ10_Rv	GGATCCTCACTGCACATGCTGTTG
cesLHA_Fw	TACTGTAAGCTTTATCCAATACGC
cesLHA_Rv	TTAAGAGTTCTCGAGTGATGTCAT
sepDXhoI_Fw	ACGGGTACTCGAGATGAACAATA
sepDKpn_Rv	ACTTATTGGTACCATTACACAATTC
tirXhoI_Fw	AAAGGATCTCGAGATGCCTATTGG
tirKpn_Rv	CTCACAGGTACCTTTAAACGAAAC
cesLXhoI_Fw	CAGAGCCTCGAGATGAATCTTTTA
cesLKpnI_Rv	ATTTAAGGGTACCTTCATGATGTC
sepLBamHIFw	ATTACGTGAGGATCCATGGCTAAT
sepLXhoIstop_Rv	AAAACTCGAGATCACATAACATCC
cesTBamHI_Fw	AAGAGAAGGATCCATGTCATCAAG
cesTKpnI_Rv	CTAATAAGGTACCTTTATCTTCCG

## Data Availability

Not applicable.

## Supplementary Materials

The following are available online at https://www.mdpi.com/article/10.3390/microorganisms9051047/s1, Figure S1. Formation of stable heterotrimeric complexes by pull-down assays. Figure S2. Solubility of His-CesL requires both SepL and SepD proteins. Figure S3. Role of the N-terminal region of SepL in the formation of the heterotrimeric complex. Figure S4. Three-dimensional structural modeling of the EPEC SepL/SepD/CesL complex. Figure S5. In vivo stability of SepL, SepD, and CesL proteins depends on each other. Figure S6. SepL membrane location is not affected in the absence of CesL or SepD.
