# Supplementary material for: CesL Regulates Type III Secretion Substrate Specificity of the Enteropathogenic E. coli Injectisome
Source: Microorganisms. 2021 May 13;9(5):1047. doi: 10.3390/microorganisms9051047 (PMC8152094; doi:10.3390/microorganisms9051047)
Supplement: Supplementary file 1 [file microorganisms-09-01047-s001.zip › microorganisms-1181615-supplementary.pdf]

CesL Regulates Type III Secretion Substrate Specificity of the Enteropathogenic *E. coli* Injectisome

Table of contents

Supplementary figures

Figure S1. Formation of stable heterotrimeric complexes by pull-down assays.

Figure S2. Solubility of His-CesL requires both SepL and SepD proteins.

Figure S3. Role of the N-terminal region of SepL in formation of the heterotrimeric complex.

Figure S4. Three-dimensional structural modeling of the EPEC SepL/SepD/CesL complex.

Figure S5. In vivo stability of SepL, SepD and CesL proteins depends on each other.

Figure S6. SepL membrane location is not affected in the absence of CesL or SepD.

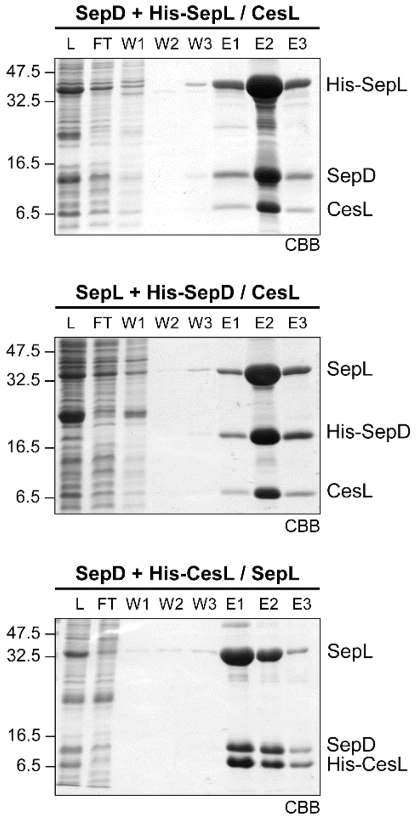

Figure S1. Formation of stable heterotrimeric complexes by pull-down assays. Ni-NTA pull-down assays using a cleared lysate obtained from *Salmonella* SJW1368 cells co-expressing **(A)** plasmids pMTBISpDpL and pMATcL, **(B)** plasmids pMTBISpLpD and pMATcL, or **(C)** plasmids pMTBISpDcL and pMATpL. The cleared lysates (L) were loaded onto Ni-NTA agarose beads and the flow-through (FT), wash (W), and elution (E) fractions were collected. All samples were visualized by Coomassie brilliant blue CBB-stained SDS-PAGE.

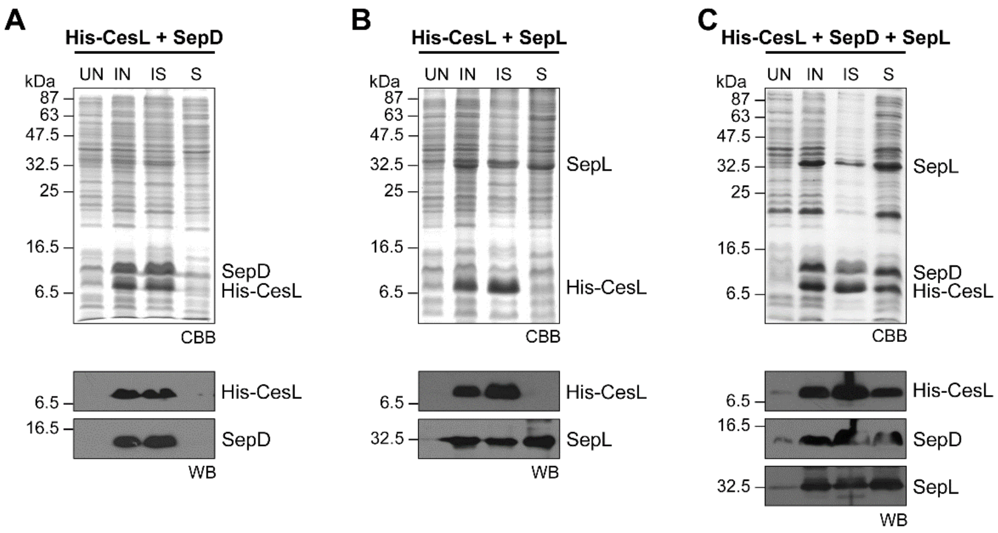

Figure S2. Solubility of His-CesL requires both SepL and SepD proteins. *Salmonella* SJW1368 cells co-transformed with plasmids **(A)** pMTBISpDcL, **(B)** pMTBISpLcL or **(C)** pMTBISpDcL and plasmid pMATpL. After protein overproduction with IPTG, bacterial cell lysates were fractionated (see Materials and methods). Samples of uninduced (UN) and induced (IN) cultures, insoluble (IS) and soluble (S) fractions were analyzed by CBB-stained SDS-PAGE (Upper panel). Immunoblots (WB) using anti-His, anti-SepL, or anti-SepD antibodies are shown in the lower panel.

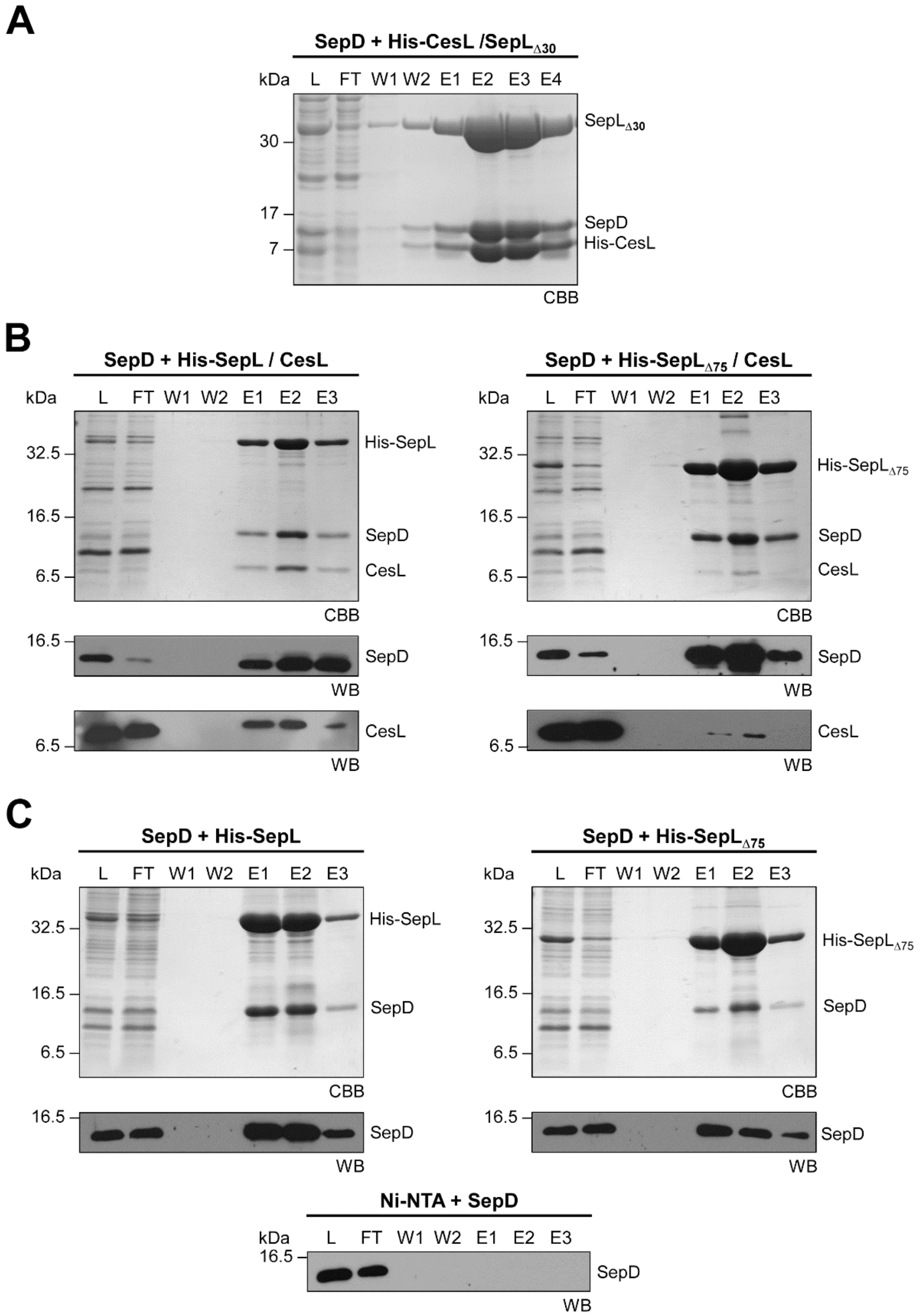

Figure S3. Role of the N-terminal region of SepL in formation of the heterotrimeric complex. Pull-downs of His-CesL, His-SepL and His-SepL$_{\Delta 75}$ were carried out using Ni-NTA agarose beads. Cleared lysates (L) from BDP cells containing the indicated recombinant proteins were loaded onto a nickel resin packaged column (see Materials and methods) and the flow-through (FT), wash (W), and elution (E) fractions were collected. All samples were visualized by CBB-stained SDS-PAGE (Upper panels).

Immunoblotting (WB) of CesL and SepD was done using antibodies against the indicated protein. SepD was loaded onto Ni-NTA agarose beads as a control.

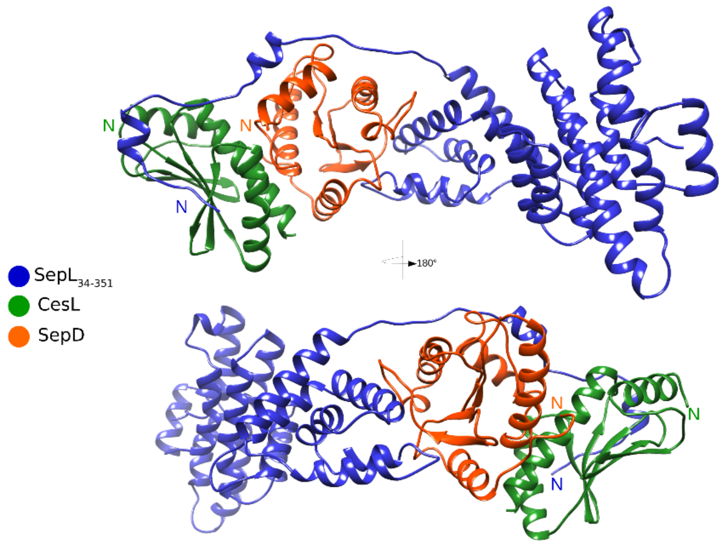

Figure S4. Three-dimensional structural modeling of the EPEC SepL/SepD/CesL complex. Ribbon representation of the structural model of SepL (blue), SepD (orange) and CesL (olive green) complex. The predicted individual models were fitted onto the structure of the *Yersinia* YopN/TyeA-YscB-SycN complex (PDB accession number 1XKP) as described in Materials and methods. Front view and side view rotated 180° about the y-axis.

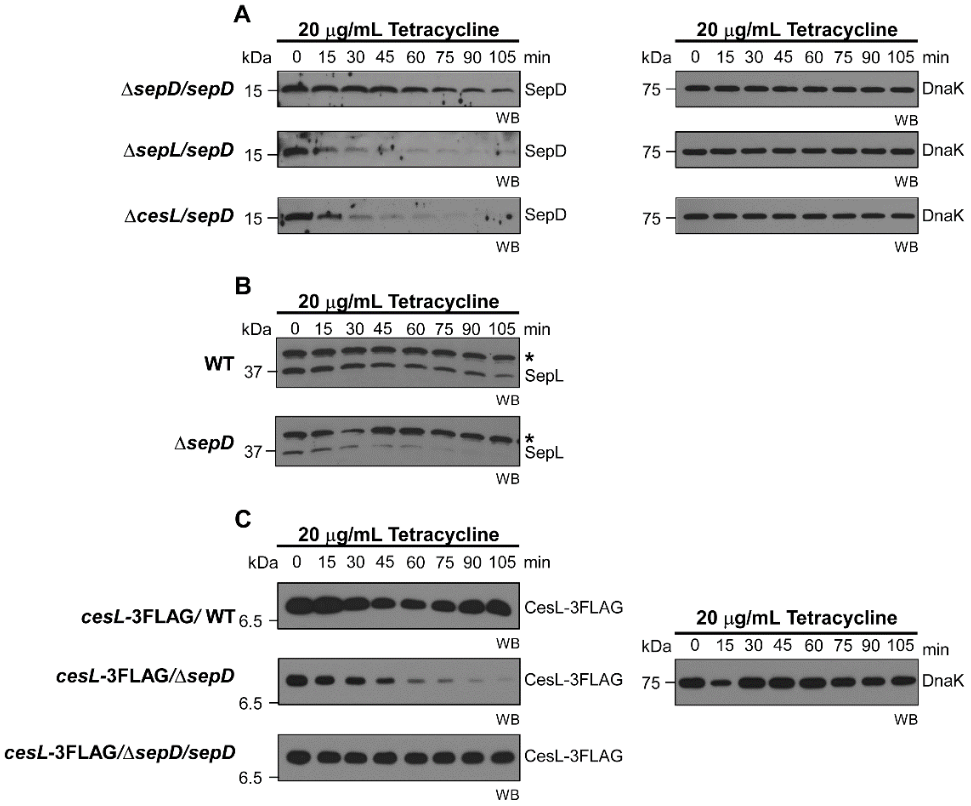

Figure S5. ***In vivo*** **stability of SepD, SepL, and CesL proteins depends on each other. (A)** EPEC Δ*sepD*, Δ*sepL*, and Δ*cesL* strains harboring the low-copy plasmid pMATpD expressing untagged SepD, were grown under T3S-inducing conditions. After halting de novo protein synthesis by addition of tetracycline, whole cell culture samples were collected every 15 min during 105 min. Samples were resolved by SDS-PAGE followed by immunoblotting using anti-SepD antibodies. **(B)** The same procedure as described in **(A)** was performed for SepL stability. Protein levels of native SepL in wild-type EPEC (WT) and Δ*sepD* strains are shown. An asterisk denotes a non-specific band that could be used as a loading control. **(C)** Protein levels of chromosomally encoded CesL-FLAG expressed in EPEC wild-type (WT), Δ*sepD*, or Δ*sepD* strain carrying the low-copy plasmid pMATpD, were assessed following the same procedure to that previously described (A). DnaK was used as a loading control. Samples were normalized according to the OD_{600} of the culture sample at the time of collection.

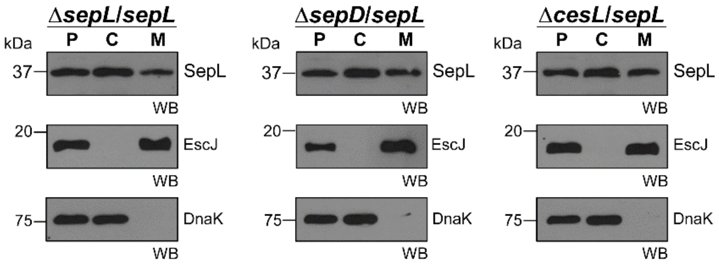

Figure S6. SepL membrane location is not affected in the absence of CesL or SepD. EPEC Δ*sepL*, Δ*sepD* and Δ*cesL* mutant strains harboring the low-copy plasmid pMATpL expressing untagged SepL, were grown under T3S-inducing conditions and fractionated into (C) cytoplasmic and (M) membrane fractions. Whole-cell lysates (P) were loaded as a control. EscJ and DnaK were used as cytoplasmic and membrane protein controls, respectively. Equal amounts of each fraction were probed using anti-SepL, anti-EscJ or anti-DnaK antibodies.
